# Induced Pluripotency: A Powerful Tool for In Vitro Modeling

**DOI:** 10.3390/ijms21238910

**Published:** 2020-11-24

**Authors:** Romana Zahumenska, Vladimir Nosal, Marek Smolar, Terezia Okajcekova, Henrieta Skovierova, Jan Strnadel, Erika Halasova

**Affiliations:** 1 Department of Medical Biochemistry, Jessenius Faculty of Medicine in Martin, Comenius University in Bratislava, 036 01 Martin, Slovakia; zahumenska13@uniba.sk (R.Z.); okajcekova1@uniba.sk (T.O.); 2 Biomedical Centre Martin, Jessenius Faculty of Medicine in Martin, Comenius University in Bratislava, 036 01 Martin, Slovakia; henrieta.skovierova@uniba.sk; 3 Jessenius Faculty of Medicine in Martin, University Hospital in Martin, Comenius University in Bratislava, 036 01 Martin, Slovakia; vladimir.nosal@uniba.sk (V.N.); marek.smolar@uniba.sk (M.S.); 4 Department of Medical Biology, Jessenius Faculty of Medicine in Martin, Comenius University in Bratislava, 036 01 Martin, Slovakia

**Keywords:** cell reprogramming, induced pluripotent stem cells, neural precursor cells, in vitro biomedical models, disease modeling, neurodegenerative disease

## Abstract

One of the greatest breakthroughs of regenerative medicine in this century was the discovery of induced pluripotent stem cell (iPSC) technology in 2006 by Shinya Yamanaka. iPSCs originate from terminally differentiated somatic cells that have newly acquired the developmental capacity of self-renewal and differentiation into any cells of three germ layers. Before iPSCs can be used routinely in clinical practice, their efficacy and safety need to be rigorously tested; however, iPSCs have already become effective and fully-fledged tools for application under in vitro conditions. They are currently routinely used for disease modeling, preparation of difficult-to-access cell lines, monitoring of cellular mechanisms in micro- or macroscopic scales, drug testing and screening, genetic engineering, and many other applications. This review is a brief summary of the reprogramming process and subsequent differentiation and culture of reprogrammed cells into neural precursor cells (NPCs) in two-dimensional (2D) and three-dimensional (3D) conditions. NPCs can be used as biomedical models for neurodegenerative diseases (NDs), which are currently considered to be one of the major health problems in the human population.

## 1. Introduction

The development of technology for induced pluripotency in 2006 by Shinya Yamanaka has opened new horizons in the field of regenerative medicine and in vitro disease modeling. A unique approach of obtaining of virtually any cell of interest from skin cells isolated from patients has not been possible in the past. Obtaining hard-to-reach tissue cells, such as neurons or cardiomyocytes, for scientific purposes and disease modeling has never been easier. Therefore, this technology provides an extremely important tool for the “disease-in-the-dish” field.

This review provides insight into the process and techniques of cell reprogramming and iPSCs with a special focus on neural differentiation, and in vitro models of NDs.

NDs are a heterogeneous group of disorders that are becoming part of the lives of an increasing number of people. Aging is considered to be one of the main risk factors associated with the increasing incidence of NDs. The growing number of ND cases leads to an enormous socioeconomic burden for both patients and their family and for society as a whole [1].

Several different types of NDs are known and characterized by different origins and molecular courses of the disease, and they also are closely linked to progressive degeneration of the function and structure of the central or peripheral nervous system [2].

Animal models provide many valuable results for understanding the overall perception and course of NDs and their molecular mechanisms. However, when the results from animal models are compared with the results from clinical studies, some key species-specific differences can be observed. These differences mean preclinical models for human diseases based on animals are not quite as accurate nor suitable [3].

Induced pluripotent stem cells (iPSCs) represent a strong tool for in vitro modeling of NDs. In the last decade, this discovery has experienced tremendous growth and success and influenced industries such as not only disease modeling but also regenerative and translational medicine and drug screening or developmental biology [4].

## 2. Stem Cells

Stem cells (SCs) are unspecialized cells that have the ability to self-renew and differentiate into different cell types [5]. These two properties predestine them to play an important role not only in regenerative medicine but also for in vitro cell and tissue modeling or drug testing. The cellular capacity of self-renewal is based on multiple mitotic divisions, during which SCs do not differentiate but preserve and increase their regenerative potential. The SCs can divide symmetrically or asymmetrically. In symmetrical division, two daughter cells arise from one parent cell. These cells are identical to the parent cell. Asymmetric division involves the generation of two daughter cells, with one cell identical to the parent cell and the second one not. Thus, the derived daughter SCs have different cellular fates. In simple terms, the asymmetrically formed daughter cell is predestined for further differentiation [6,7]. There are several SC types and they can be classified according to their different degrees of potency as totipotent, pluripotent, multipotent, oligopotent, or unipotent [5,8].

## 3. iPSCs

Induced pluripotency is led by the controlled expression of certain transcription factors in adult somatic cells that are already differentiated and non-pluripotent [9]. This kind of nuclear reprogramming is defined as a change in the differentiation properties of mature-cell characteristics for the undifferentiated embryonic state [10]. The first iPSCs were created by Shinya Yamanaka in 2006 [11] following many studies in the six decades that preceded this discovery. Takahashi and Yamanaka initially reprogrammed adult mouse skin fibroblasts to cells very similar to embryonic stem cells (ESCs). These fibroblasts were transduced with retroviral vectors containing 24 selected genes coding transcription factors (TFs) and selected on the assumption that they maintain pluripotency and self-renewal ability characteristic of ESC biology. Among the selected TFs were genes encoding octamer-binding transcription factor 4 (Oct4), sex-determining region Y-box 2 (Sox2), cellular myelocytomatosis oncogene (c-Myc), and Kruppel-like factor 4 (Klf4), which are now known as Yamanaka factors. Adult differentiated somatic cells were reprogrammed within two weeks and behaved like ESCs, i.e., pluripotent self-renewing cells with the ability to differentiate into different cell types [10]. One year later, the experiment was reproduced using human fibroblasts [12,13]. In 2012, this discovery was awarded the Nobel Prize in Physiology or Medicine. However, this would not be possible without the discovery that specialized somatic cells have the same genetic information as early embryonic cells. This fact has been well demonstrated by experiments known as somatic cell nuclear transfer (SCNT), where terminally differentiated cells demonstrate the totipotent potential of their genome [14,15,16,17,18]. Additionally, the techniques that made it possible to obtain, culture, characterize, and study pluripotent SCs and the discovery of very important proteins—transcription factors involved in the control of the transcription process and, thus, in determining cellular fate—were also essential [9].

A decade has passed since the first mention of iPSCs, and iPSCs have markedly revolutionized the field of regenerative medicine. Significant progress has been made in the area of reprogramming techniques, culture methods, safety, and manipulation of iPSCs. This was of interest because the initial methods of iPSC generation were either ineffective or results were difficult to reproduce. In addition, a better alternative to the use of transcription factors, which are classified as potent oncogenes, was required [19,20]. Cells prepared by reprogramming techniques are used in several areas of research because iPSCs offer the possibility of preparing any type of cell line with well-defined cell properties. The unique properties of iPSCs predetermine them to serve as a tool for understanding the pathogenesis and molecular changes associated with many diseases [21,22], their genome can be edited with the intention of repairing/inducing damaged genes [23] while specific reporter, knockout, or isogenic cell lines are generated. These predictive models can be used for disease modeling and drug testing [24], and they have the potential to reduce or even replace animal models [22].

Thirteen years after the discovery of iPSCs, thanks to the great interest of the scientific community, we are now achieving these goals, primarily those based on in vitro conditions. Clinical use is partially limited, so research relies on the optimization of reprogramming techniques, conversion of cell fate, and advances in tissue engineering, safety, or efficacy [25].

### Biological Characterization, Benefits, and Limitations of iPSCs

From the beginning, it has been assumed that the use of iPSCs is moving towards two different pathways: first as a tool for regenerative medicine and clinical practice [26] and, second, as a tool for disease modeling and drug screening [27]. In both cases, the advantages and limitations of iPSCs must be considered. Therefore, when looking at possible applications of iPSCs, it is necessary to know their biology at the molecular, genetic, epigenetic, and morphological levels (Table 1) and, in addition, know and reveal their limits.

Not all tissues in the human body are equally easy to obtain. Some tissues (such as nerve cells) are poorly available and are difficult to biopsy for further research. In this case, a biopsy is very invasive and painful for patients. In this regard, a significant revolution was made after the discovery of iPSCs, which offer an alternative source of cells. The aforementioned differentiation ability of iPSCs potentially allows them to be used to create any type of required cell, even those of tissues that are difficult to access [35]. As already mentioned, this possibility is considered to be one of the biggest benefits of iPSCs.

When preparing iPSCs, certain strict rules must be followed, so their ability to self-renew, differentiation efficiency, and their subsequent application, is not affected. Several key points in regard to that process are highlighted here:

- Selection of a suitable microenvironment, extracellular matrix, and proteins;
- Selection of appropriate reprogramming factors;
- Selection of suitable growth factors;
- Selection of appropriate differentiation factors.

Overcoming these barriers requires the integration of knowledge and technology from a variety of scientific areas, including cellular and molecular biology, biomedicine, bioengineering, and biophysics [35,36]. Working with iPSCs involves processes such as somatic cell preparation, cell reprogramming, expansion and culturing, and differentiation and their development for future applications. As in other areas or fields, some issues and limitations connected with iPSCs exist. A summary of the main advantages and disadvantages of human iPSCs (compared to human ESCs) is shown in Table 2. Although it is still necessary to optimize techniques and processes for cell cultivation, generation, maintenance, and differentiation so that their use is safe, efficient, and cost-effective, their potential in a variety of applications indicates a promising future for therapeutic use [37].

## 4. Reprogramming Process

**Figure 1:**
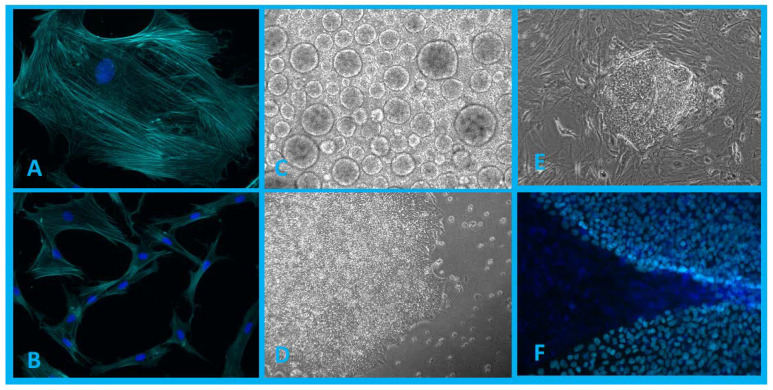
(**A**,**B**)—Population of human dermal fibroblasts isolated from patient biopsy (blue, DAPI; cyan, phalloidin). (**C**)—Embryoid bodies formed in 3D cultivation conditions. (**D**,**E**)—iPSC colonies in 2D cultivation conditions. (**F**)—iPSC colonies grown on a layer of mouse embryonic fibroblasts (MEFs) (blue, DAPI; cyan, expressed transcription factor Nanog). Authors’ own images.

Reprogramming cells into a pluripotent state is a dynamic process characterized by morphological changes (Figure 1) and changes at all important cellular levels, including gene expression and the proteome, epigenome, and metabolome [40].

Several studies have shown that the process of reprogramming somatic cells to the iPSC stage is still incomplete, and iPSCs exhibit a relatively high number of aberrant epigenetic traits that hinder their further possible application. Epigenetic remodeling involves processes of genomic change, DNA methylation, histone modifications, and X-chromosome reactivation. Therefore, further efficient generation and, consequently, safe differentiation of iPSCs requires knowledge of the mentioned molecular mechanisms and their subprocesses. It is also necessary to continuously improve and optimize the protocols in order to achieve the best and most effective results [41,42,43].

There are many ways and techniques to reprogram somatic cells to the stage of induced pluripotency. The three most important parameters that influence the reprogramming process are:

- Selection of the appropriate type of somatic cells;
- Selection of appropriate reprogramming factors and their combination;
- Selection of a method and suitable way for delivering reprogramming factors to the somatic cells.

### 4.1. Selection of the Appropriate Type of Somatic Cells

A suitable cell type is preferably one that is readily accessible and with relatively high and efficient reprogramming kinetics as these attributes vary depending on the cell type. Keratinocytes, for example, were reprogrammed twice as fast as human fibroblasts under the same conditions [44,45]. Furthermore, there is a possibility to use cells that are less invasive to obtain, e.g., blood cells [46] or epithelial cells isolated from urine [47,48], which can be classified as easily accessible, biological waste material.

The selection of cells suitable for reprogramming and subsequent differentiation has been the subject of many discussions because different cells reprogram with different sensitivities [48]. According to several studies, the differentiating capacity of iPSCs is influenced by the epigenetic memory of the original outgoing somatic cells [49,50] while other researchers suggested that the differentiation capacity of iPSCs does not depend on the type of starting cell but on the degree of DNA methylation during the reprogramming process [51]. However, the research results, overall, seem to favor the first statement [52].

### 4.2. Selection of Appropriate Defined Re-Programming Factors and Their Combination

The process of iPSC derivation requires the introduction of exogenous TFs into somatic cells. TFs control the rate and efficiency of the transcription of genetic information from DNA to RNA. Each transcription factor has a specific role in one or more molecular signaling pathways. Factors must be positively or negatively regulated to induce the reprogramming and expression of genes in the right cell, at the right time, and in the right amount (Table 3) [44]. These molecular factors play an especially important role in determining cell specialization while maintaining cell identity and also helping to control cell fate [53,54]. For the first set of reprogramming experiments, the TF combination known as OSKM—Oct4, Sox2, Klf4, and c-Myc—was used [11]. However, Oct4 is associated with cervical cancer, Sox2 is highly expressed in melanoma cells, Klf4 and c-Myc are involved in cell proliferation, and c-Myc is also a well-known protooncogene. Therefore, it was more than necessary to look for new possibilities and combinations of factors whose application would be safer.

**Figure 2:**
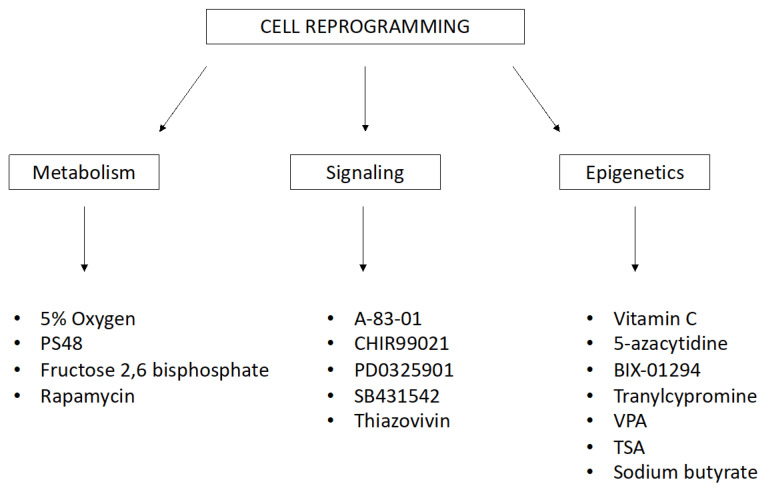
Small molecules for reprogramming and transdifferentiation that affect biochemical and molecular processes in cells. (PS48—Allosteric Phosphoinositide-Dependent Protein Kinase-1 (PDK1) agonist, VPA—Valproic Acid, TSA—Trichostatin A).

The variability in reprogramming efficacy has also been demonstrated using the same method. Small natural or synthetic chemical molecules are readily available. They are able to increase the efficiency of reprogramming and differentiation by inhibiting or inducing particular cellular processes and epigenetic and signaling pathways. These small chemical molecules work in combination with reprogramming transcription factors, but they can serve as functional replacements. The list of these stimulating treatments, with diverse background and scope, is extensive. Small molecules for reprogramming and transdifferentiation (Figure 2) include the following: 5% oxygen, 5-azacytidine, A-83-01, CHIR99021, PD0325901, SB431542, sodium butyrate, valproic acid (VPA), vitamin C, thiazovivin, and tranylcypromine [61,62,63,64].

### 4.3. Selection of a Method for Delivery of Reprogramming Factors to the Somatic Cells

Despite the development of new procedures, iPSC derivation technology remains quite complicated. The greatest efficacy has been achieved with the use of viral vectors that, on one hand, are relatively risky for clinical application. New non-viral, non-integrating techniques are relatively safe but the level of iPSC derivation efficiency is slightly lower compared to that of viral techniques [65]. The great improvement came with the development of third-generation reprogramming techniques. Synthetic and self-replicative RNA currently appears to be the safest and most efficient reprogramming method [66]. Another improvement was the discovery of a growth-factor-free culture system by Yasuda et al. Yasuda with coworkers prepared a chemically defined culture medium using only three chemical compounds, no growth factors, and a minimum of recombinant proteins compared to other commercially available culture media. iPSCs cultured in defined, xeno-free conditions showed promising results at the genetic (karyotype) and biological level (immunocytochemistry). This was a significant contribution to the development and the use of iPSCs [67].

## 5. Cell Reprogramming Techniques

**Figure 3:**
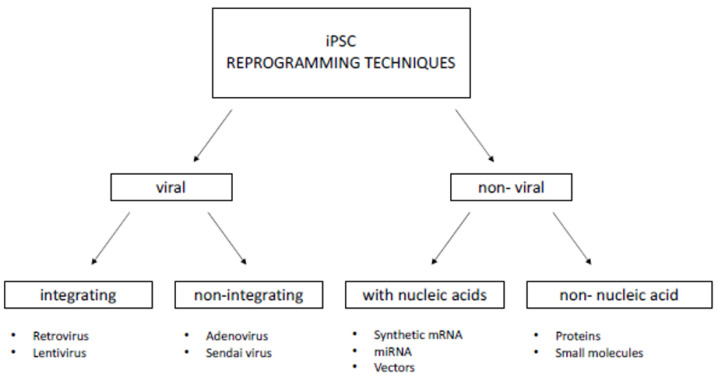
The most commonly used iPSC reprogramming approaches.

In this section, we will discuss the most well-known, exploited, and applied first- and second-generation reprogramming techniques. Methods can generally be classified into two groups: viral and non-viral. The viral approach consists of introducing transcription factors into cells via transfection with non-cellular organisms that can integrate/not integrate into the host genome. The non-viral approach involves introducing factors into cells through nucleic acids or their translated products, i.e., proteins. A diagram of the most commonly used reprogramming techniques is shown in Figure 3.

The choice of a suitable reprogramming method depends primarily on the targets and desired direction of the research. It is also influenced by the efficiency of the technique, the reprogramming capability of the current cell type, and the footprint to be left with iPSC generation [62].

Reprogramming itself lasts 14 to 56 days, depending on the method and protocol. In general, when we compare methods and their efficiency of iPSC derivation, the use of synthetic mRNA shows the best outcomes and is followed by viral approaches (retro/lenti/Sendai virus). However, when we consider other parameters such as the cost, preparation of materials (retro/lentivirus), procedure of delivery (viral methods), and removal of exogenous factors (Sendai virus and synthetic mRNA), choosing a suitable technique is challenging [65,68,69].

Critically controlling the efficacy of cell reprogramming consists mainly of evaluating the expression of specific intracellular and surface markers, morphological analyses, and in vivo teratoma assays [70].

## 6. Reprogramming and Metabolic Shift

iPSCs have unique metabolic properties that are similar to cancer cells, especially their upregulated proliferation, glycolysis, and telomerase activity. The metabolism of iPSCs ensures the maintenance of cellular homeostasis, pluripotency, and self-renewal capabilities and, at the same time, ensures a rapid response during cell differentiation [71]. Understanding the mechanisms underlying iPSC derivation is necessary to maintain the sustainability, quality, and safety of using iPSCs [71,72]. In addition to the epigenetic, morphological, and transformational changes that occur in cells during the reprogramming process, a significant metabolic shift may also be involved. Among the biochemical processes occurring in cells, oxidative phosphorylation is the most markedly suppressed, while the glycolytic pathway is promoted. Glycolysis produces ATP, even in the presence of oxygen, and it is linked to other biochemical pathways that provide the building blocks for the synthesis of nucleic acids, non-essential amino acids (NEAA), and lipids [73,74,75].

There is also remarkable remodeling of mitochondria, the energy centers of cells that rejuvenate and change their morphology (Figure 3), size, and localization to be closer to the cell nucleus. At the same time, the expression of mitochondrial proteins is also altered which, in turn, supports the hypothesis that functional mitochondrial changes and upregulation of glycolysis are necessary before the induction of pluripotent genes [71,76,77]. Cellular metabolism and biochemical pathways are closely linked to regulatory mechanisms at both transcriptional and post-transcriptional levels; therefore, even slight changes are subsequently reflected in the interpretation of outcomes and results [71].

## 7. Neural Differentiation of iPSCs

Differentiation is the most important step for the successful application of iPSCs in diagnostics, disease modeling, or regenerative medicine. When cells are transplanted prior to differentiation, iPSCs form teratomas under in vivo conditions. iPSC generation is possible using different approaches, and their effect reflects their differentiation potential. Therefore, the choice of differentiation strategy is very important [78].

The preparation of neural precursor cells (NPCs) and nerve cells like motor neurons (MNs) derived from human iPSCs leads to the emergence of cell models that offer features consistent with human physiology and genetics. At the same time, they are not subject to ethical issues, cross-species variability, or future immunological rejection after implantation in personalized treatment. Depending on what particular type of neural cells are needed for specific NDs, the protocols vary accordingly. The protocols also reflect the requirements for further use of neurodegenerative models that can serve in primary molecular research, drug testing and screening, repair of damaged genes, or can be further translated into clinical practice [79].

For successful cell differentiation, it is necessary to ensure the interplay of several factors such as timing, appropriate combination and concentration of growth and differentiation factors, small molecules, biophysical factors, and the environment to activate appropriate signaling pathways and increase gene expression.

During in vivo neural development, a number of molecular changes and mechanisms are activated. This is manifested by the development of different types of neurons, their location, cellular connections, morphology, expression profiles, and their function or potential to generate electrical impulses. In vitro cell reprogramming and differentiation is a lengthy and demanding process. The production and validation of a new functional neuronal cell line from original fibroblasts may take four to six months. Culture media used in differentiation into NPCs and MNs are generally treated with well-known additives at the desired concentration, e.g., NEAA, laminin, brain-derived neurotrophic factor (BDNF), neurotrophin-3 (NT-3), Y-27632, L-ascorbic acid (LAA), epidermal growth factor (EGF), basic fibroblast growth factor (bFGF), B27, N-2, CHIR99021, SB431542, retinoic acid (RA), sonic hedgehog signaling molecule (SHH), and other substances [79,80,81]. Most of them are summarized in Table 4. This is a suitable combination of cell-fate-determining factors that are responsible for overall maturation and full cell line differentiation [82].

## 8. NDs

Among the health problems occurring in modern society, neurological disorders are one of the most serious. These may be of a neuropsychological or neurodegenerative character. The origin and occurrence of these diseases are ambiguous and involves the interplay of several genetic, epigenetic, and environmental factors. After diagnosing the disease, problems can persist for decades, and they often have a progressive course [83].

NDs, as the name implies, cause degeneration or death of neural cells. This group of incurable diseases with a strong progressive character is manifested as ataxia or dementia in patients. The prevalence of their occurrence increases every year in connection with prolonged life expectancy [84]. In the coming decades, this will be greatly reflected by the socioeconomic burden to patients, their family circle, and social and healthcare facilities.

ND is often seen in a broader context only as a group of diseases that affect different areas of the brain and present different manifestations, pathology, and molecular etiology. However, with a closer look, one can observe certain common characteristics and the same players that repeatedly emerge at the molecular and genetic levels. The most common factor is protein and peptide aggregation, whether cytosolic or nuclear, in individual regions of the central or peripheral nervous system [85] leading to neurovascular dysfunction [86].

Gan et al. conducted [85] a comprehensive review of current knowledge at the time, mentioning the involved pathways and pathology of NDs, dysfunction at the mitochondrial and lysosomal levels, changes in autophagy, synaptic toxicity, the involvement of stress granules, and other mechanisms. Disruption of neural cell homeostasis, which interplays with aging, genetic variations at the level of single-nucleotide polymorphisms (SNPs), and epigenetic changes, ultimately encourages activation of the immune response and progression of neurodegeneration. Major diseases include Alzheimer’s disease (AD), Parkinson’s disease (PD), amyotrophic lateral sclerosis (ALS), Huntington’s disease (HD), spinal muscular atrophy (SMA), spinocerebellar ataxia (SCA), motor neuron diseases (MNDs), and frontotemporal dementia (FTD) (Table 5).

The search for suitable therapeutic treatments for these incurable diseases lies in the development of appropriate model systems [87].

## 9. In Vitro Models of NDs

The establishment of models relevant to NDs is difficult, not only financially but also from biotechnological and bioengineering aspects [79].

Many NDs have a heterogenous origin due to a combination of variant alleles [88]. Although animal in vivo models and, in particular, rodent models, provide valuable tools for neural research, the possibilities for their translation into clinical practice are quite limited as they are more suitable for modeling single-gene diseases [88,89].

In general, a large number of animal studies have been performed to investigate NDs. During the testing of new drugs, many animal trials appeared to be successful, but due to interspecies differences and inadequate modeling, they failed in numerous clinical trials [90,91]. This has prompted the search for new, more efficient options.

Investigations of post-mortem neural tissue at both molecular and macroscopic levels have failed to accurately explain the complex dynamics of NDs [92].

When functional neurodegenerative conditions are modeled, great emphasis is placed on the rapid production, quantity, and quality of neural cells, which must be able to handle the simulation of conditions throughout the ongoing experiment [93]. The main basis of each relevant experiment is the generation of mature and functional neural cells, with a mature phenotype and sufficiently silenced expression programs of the original cell population [94].

iPSCs play a prominent role in cell modeling of NDs. However, significant progress in the field has been made after the introduction of a new, innovative way of culturing cells: three-dimensional (3D) organoids [95].

## 10. 3D Cultivation as a Promising Approach in Creating Models of NDs

Two-dimensional (2D) cell models are the most widely used platform for modeling disease using iPSCs [83]. They are attractive mainly due to the relative simplicity of cultivation, optimized conditions, and low financial burden. However, they do not sufficiently reflect the real 3D environment; they lack oxygen, nutritional, or waste gradients; the architecture of these cultures does not provide interactions between cells or between cells and the extracellular matrix (ECM). These facts are reflected in the distorted biochemical and biophysical processes that affect the bioactivity and expression profile of cells [96]. The cells are cultured under well-defined conditions, which vary depending on the particular cell type and line. In general, it is necessary to provide a suitable environment. Optimal pH, temperature, osmotic pressure, culture vessel, and nutrients, including culture medium, amino acids, carbohydrates, vitamins, minerals, growth factors, hormones, and an optimal ratio of O_2_ and CO_2_ are just a short list of the essential requirements. 3D cultures are invaluable prediction tools in simulating in vivo environmental conditions. In general, they can be divided into two main groups, namely 3D cell cultures grown with scaffold support, better known as scaffold-based cultures, and 3D cell cultures grown without scaffold support, better known as scaffold-free cultures [97]. Their architecture models real tissue conditions. 3D cultures thus represent models in which cells are able to interact with each other and also interact with the ECM [98]. Although there is no universal 3D microenvironment for a particular area of research, it is known that 3D culture conditions designed in a specific way are reflected in cellular processes such as growth, migration [99], proliferation [100], differentiation or gene expression, and protein production [101]. Interesting results were obtained by Song et al., who investigated the control of NPCs properties using 2D and 3D conductive polymer scaffolds. They found that the expression of genes related to proliferation and metabolic pathways was altered due to the interplay between the physical nature of the microenvironment and the application of the electric field [102].

The advantage of 3D cultures compared to 2D systems is that they can better predict efficacy, sensitivity, or toxicity of drugs and, at the same time, eliminate differences between species (when compared to animal models), which sometimes distorts the interpretation of preclinical results (Table 6) [103]. One of the main disadvantages that can be attributed to their further limitations is insufficient vascularization as well as the not yet solved inconsistency and variability of individual batches of organoids; in other words, not all organoids produce cells of the same quality and quantity [104,105]. However, 3D cultures are still not widely used and are in the exploration and testing phase. There is no universal 3D matrix, so every new substrate offers new conditions and cultivation possibilities for cells, which will be reflected in their bioactivity; therefore, the results may not always be reproducible. Nevertheless, this cultivation technology is an increasingly attractive alternative in order to study a wider spectrum of cellular processes and possibilities in vitro [96].

The iPSC-derived 3D neural models prepared by this technological process open up a new platform in which it is possible to ask questions and seek answers regarding the pathogenesis of neurodevelopmental and neuropsychiatric disorders [92,106].

## 11. 3D Brain Organoids—The Future of In Vitro Modeling?

The most well-known 3D neural models, recapitulating demanding and complex conditions in vivo, are the so-called mini-brains. These models consist of a heterogeneous self-assembled population of cells suitable for long-term culture [106].

In combination with the molecular genetic technique known as clustered regularly interspaced short palindromic repeats and CRISPR-associated protein 9, in short, CRISPR/Cas9, which allows targeted genetic manipulation by altering, cutting, or inserting parts of genetic information, the field of action has reopened, and we are again one step closer to gaining success in clinical practice [107].

**Figure 4:**
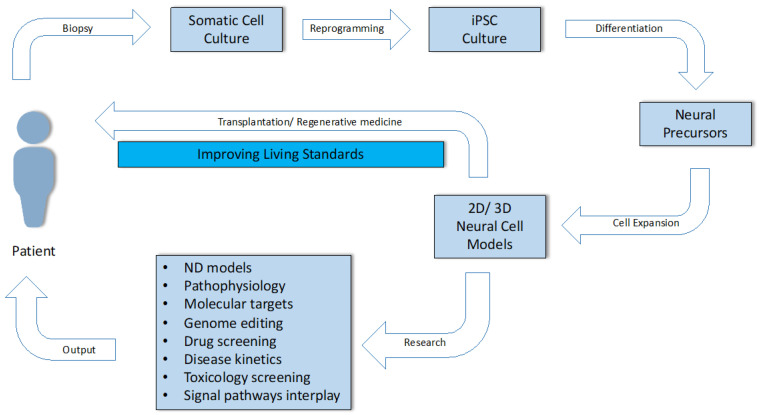
Use of the somatic cells of an adult patient through the stages of culturing, reprogramming, differentiation, biological characterization, in vitro modeling of disease, drug testing, and the possibility of their use in clinical practice and personalized regenerative medicine.

Qian and colleagues were able to prepare a heterogeneous population of cells consisting of the neurons needed to form all six layers of the human brain [108]. Comparisons of biological development and organization of the brain, in vivo and in vitro, would therefore be very similar and realistic, especially in terms of early stages at the morphological, gene, and temporal levels. Later stages show subtle differences, such as the absence of vascularization and diversity of neural precursors and cortical wall size [109]. Although there are still quite a number of issues in this area, the number of studies grouping the attractive potential of iPSCs, 3D cultivation, and in vitro ND modeling is increasing every year. The use of 3D neural models has an irreplaceable role in investigating the pathophysiology of NDs, in observing the dynamic expression of risk factors, biomarkers, up-/downregulated signaling pathways and their interplay, and drug screening (Figure 4).

## 12. Conclusions

The technology of induced pluripotency caused a small revolution in the field of disease modeling and will probably soon change the field of regenerative medicine as well. iPSCs offer a new approach in disease modeling, representing a relatively readily available, unique source of cells originally derived from terminally differentiated somatic cells (most commonly fibroblasts) that have undergone reprogramming. The cells have altered properties and regained their ability to self-renew and differentiate, and undergone a significant metabolic shift.

Although not yet ready for multispectral clinical use, the iPSCs continue to show their huge potential for disease modeling, especially in the case of diseases with hard-to-get samples, as NDs. As life expectancy increases, the prevalence of NDs, which are characterized by progressive and selective loss and dysfunction of nerve cells, also increases every year. iPSCs have the potential to accelerate the research progress by providing personalized in vitro cell models.

This is quite important as the molecular and genetic background of individual diseases is variable, as is the spectrum of clinical manifestations. Until recently, the study of their pathophysiology and pathology was largely limited, mainly due to the difficulty of accessing nerve tissue, insufficient post-mortem analyses, and interspecies differences between animal models and humans. Although there has been a significant shift in both laboratory and clinical practice, the current prognostic biomarkers and treatments are insufficient. In vitro modeling of NDs through an intermediate step such as reprogramming and induced pluripotency offers an advantage because the primary source of cells may come from a patient with a certain type of ND. Such cells, after differentiation into neural precursors, contain the genetic information about the origin of the disease as well as an identical phenotype. Moreover, a new trend with promising results is represented by in vitro 3D modeling of NDs. Cell aggregates, also known as organoids, are formed in 3D conditions. During differentiation, NPCs form a heterogeneous mixture, reminiscent of in vivo neural architecture, where concentration gradients, cell–cell communication, and cell–extracellular matrix interactions are present. The cells subsequently respond to the optimal conditions thus created by altered expression and regulation. The primary line serving as a model of a particular ND can be further characterized by a variety of molecular and biological techniques, tested for sensitivity to a variety of drugs, and is capable of further application in clinical practice and personalized regenerative medicine. Until then, it is necessary to improve and unify cultivation procedures, discover new prognostic and advanced biomarkers, and identify molecular and genetic targets involved in the origin and development of disease. iPSC technology will be an important part of that process.

## Figures and Tables

**Table 1:** Biological properties of iPSCs.

Biological Properties of iPSCs		References
**Self-renewal**	Necessary for the maintenance of iPSCs	[28]
**Differentiation potential**	Differentiation of cells derived from three germ layers Formation of teratoma in vivo	[29]
**Genetic analysis**	Diploid karyotype Transgene silencing after reprogramming	[29]
**Epigenetic analysis**	DNA demethylation of key genes for pluripotency DNA methylation of genes determining cell type	[29]
**Markers of pluripotency**	Alkaline phosphatase analysis Surface markers: stage-specific embryonic antigen 4 (SSEA4), Tumor-related antigen 1-81 (TRA 1-81), TRA-1-60, cluster of differentiation CD30 Intracellular markers: NANOG, OCT4, SOX2, c-MYC	[29,30,31]
**Morphology**	Flat-shaped cell colonies (2D condition)	[29]
	Spheroids (in bioreactors)	[32]
	Embryoid bodies	[33]
	Monolayers	[34]

**Table 2:** Comparison of the main advantages and disadvantages of human iPSCs and ESCs [4,38,39].

		iPSCs	ESCs
**Pros**	Ethical issues	no	Depends on the laws in each country
	Differentiation of all cell types from three germ layers	yes	yes
	Availability	Easy	Difficult, limited to blastocysts (four to five days post-fertilization)
	Blood group compatibility in personalized therapy	yes	Depends on the hESC cell line
	HLA histocompatibility in personalized therapy	yes	Depends on the hESC cell line
	Disease modeling ability	high	possible
	Drug development and testing	yes	yes
**Cons**	Financially expensive	Comparable	Comparable
	Reprogramming efficiency	Depends on the reprogramming technique	Not applicable
	Immunosuppression in personalized therapy	Depends on the reprogramming technique, viral induction of iPSCs would likely induce the rejection of grafted cells, whereas non-integrative does not	yes
	Risk of teratoma formation in personalized therapy	yes	yes
	Risk of mutagenesis in personalized therapy	yes	yes

**Table 3:** Overview of the most frequently used reprogramming factors for iPSC derivation. Revised by [44,55].

Reprogramming Factors	Main Function or Effect	References
c-Myc	Maintaining the capacity of pluripotency and self-regulation	[11]
E-cadherin	Suppressor, replacement of Oct4	[56]
Glis1	Increased pluripotency, effect on Wnt/β-catenin pathways; PI3K; TGF	[57]
Klf4	Maintaining pluripotency and self-regulation	[11]
Lin28	maintenance of pluripotency, translational enhancer, Let7 inhibitor	[58]
Nanog	Maintaining pluripotency and self-regulation	[58]
Oct4	Maintaining pluripotency and self-regulation	[11,59]
Sox2	Maintaining pluripotency and self-regulation	[11,60]

(Glis1—Glis Family Zinc Finger 1, Lin28—Protein Lin-28 Homolog A, PI3K—Phosphoinositide 3-Kinase, TGF—Transforming Growth Factor).

**Table 4:** The most commonly used additives that promote differentiation of iPSCs into neural precursor cells (NPCs) and, further, to motor neurons (MNs).

NPC Differentiation	Motor Neuron Maturation
Neural induction medium 10% KnockOut serum NEAA LAA SB431542 CHIR99021 Dorsomorphin bFGF EGF B27 1% penicillin/streptomycin	Neural induction medium B27 N-2 NEAA LAA RA SHH SAG Purmorhamine CNTF BDNF NT-3 GDNF

SAG—Smoothened Agonist, CNTF—Ciliary Neurotrophic Factor, NT-3—Neurotrophin-3, GDNF—Glial Cell-Derived Neurotrophic Factor.

**Table 5:** Summary of select NDs [85].

NDs	Laden Region of Brain	Clinical Expression and Problems
**AD**	Cerebral cortex	Guidance function
	Basal ganglia	Motions, remuneration
	Thalamus	Perceptions
	Hippocampus	Memory
**HD**	Cerebral cortex	Guidance function
	Basal ganglia	Motions, remuneration
**FTD**	Cerebral cortex	Guidance function
	Basal ganglia	Motions, remuneration
	Thalamus	Perceptions
**PD**	Basal ganglia	Motions, remuneration
	Thalamus	Perceptions
**SCA**	Cerebellum	Motions, stability
	Brain stem	Basic features
**ALS**	Brain stem	Basic features
	Spinal cord lamina IX	Muscle response

**Table 6:** Summary of the most common advantages and disadvantages of 3D cultivation [96,105].

3D Models	
Pros	Cons
Modeling of difficult to access tissues	Many non-uniform protocols
Monitoring developmental stages	Many different materials
Diversity of cell types	Great variability of results
Interaction between cell types	Insufficient vascularization
Identical genetic background	Interaction
Possibility of genetic manipulation	
Easy handling	
Spatial organization	
Tailor-made microenvironment	

## Abbreviations

AD	Alzheimer’s Disease
ALS	Amyotrophic Lateral Sclerosis
BDNF	Brain Derived Neurotrophic Factor
bFGF	Basic Fibroblast Growth Factor
CD	Cluster of Differentiation
c-Myc	Cellular Myelocytomatosis Oncogene
CNTF	Ciliary Neurotrophic Factor
ECM	Extracellular Matrix
EGF	Epidermal Growth Factor
ESC	Embryonic Stem Cells
FTD	Frontotemporal Dementia
GDNF	Glial Cell-Derived Neurotrophic Factor
Glis1	Glis Family Zinc Finger 1
HD	Huntington’s Disease
iPSCs	Induced Pluripotent Stem Cells
Klf4	Kruppel-Like factor 4
LAA	L-Ascorbic Acid
LD	Linear Dichroism
MNs	Motor Neurons
MNDs	Motor Neuron Disease
NDs	Neurodegenerative Disease
NEAA	Non-Essential Amino Acids
NPCs	Neural Precursor Cells
NT-3	Neurotrophin 3
Oct4	Octamer Bunding Transcription Factor 4
OSKM	Group of Reprogramming Factors: Oct4, Sox2, Klf4, c-Myc
PD	Parkinson’s Disease
PI3K	Phosphoinositide 3-Kinase
PS48	Allosteric Phosphoinositide-Dependent Protein Kinase 2 (PDK1)
RA	Retinoic Acid
SAG	Smoothened Agonist
SCA	Spinocerebral Ataxia
SCNT	Somatic Cell Nuclear Transfer
SCs	Stem Cells
SHH	Sonic Hedgehog Signaling Molecule
SMA	Spinal Muscular Atrophy
SNPs	Small Nuclear Polymorphisms
Sox2	SRY (Sex Determining Region Y)-box 2
SSEA4	Stage-specific Embryonic Antigen 4
TFs	Transcription Factor
TRA-1-81	Tumor-Related Antigen 1-81
TRA-1-60	Tumor-Related Antigen 1-60
TSA	Trichostatin A
VPA	Valproic acid
